# “Emerging Technologies and Medical Countermeasures to Chemical, Biological, Radiological, and Nuclear (CBRN) Agents in East Ukraine”

**DOI:** 10.1186/s13031-020-00279-9

**Published:** 2020-05-08

**Authors:** Sonny S. Patel, Robert M. Grace, Patrick Chellew, Mykola Prodanchuk, Olha Romaniuk, Yuriy Skrebets, Sergii A. Ryzhenko, Timothy B. Erickson

**Affiliations:** 1 Fellow, NIH Fogarty Global Health Humanitarian Scholar, Harvard University, Cambridge, USA; 2 USIP-Minerva Peace Scholar, United States Institute of Peace, Graduate Research Fellow, Program on Negotiation, Harvard Law School, Cambridge, MA USA; 3 Medical ATO, Kyiv, Ukraine; 4 L.I. Medved’s Research Center of Preventive Toxicology, Food, and Chemical Safety, Ministry of Health of Ukraine, Kyiv, Ukraine; 5 ODC U.S. Embassy, Kyiv, Ukraine; 6 Regional Clinical Hospital, I.I. Mechnikov, Dnipro, Ukraine; 7 Regional Clinical Hospital, I.I. Mechnikov, Dnipro, Ukraine; 8 Brigham Health, Harvard Medical School, Harvard Humanitarian Initiative, Boston, USA

**Keywords:** Ukraine, Chemical, biological, radiological, and nuclear, Hybrid warfare, Cyberattacks, Train-the-trainer, Emergency preparedness and response

## Abstract

Since 2014, Ukraine has been beset by an armed conflict with international and internal dimensions. The nature of this conflict is multidimensional, and disaster preparedness and response in this context must be as well. Health experts from Ukraine, the United States of America, United Kingdom, Czech Republic, and Norway convened for an educational event in Dnipro, East Ukraine on November 11-15, 2019. At the event, “Emerging Technologies and Countermeasures to CBRN Agents: Advanced Training Response to Conflict and Security Challenges in East Ukraine,” over 1,000 participants participated in panel discussions, didactic lectures, and an advanced training on various dimensions of disaster response. This report provides an overview of the key discussions and outcomes of the event.

## Introduction

Since 2014, Ukraine has been beset by an armed conflict with international and internal dimensions. Russia now occupies one portion of the country (Crimea) and Russian-aligned separatists control others (portions of the Donetsk and Luhansk oblasts in eastern Ukraine). The conflict has become “frozen” with a relatively stable contact line dividing government-controlled from non-government-controlled territory and violence persisting at a relatively low level. Nevertheless, the humanitarian consequences remain dire. The United Nations estimates that 3.5 million people remain in need of humanitarian assistance [1]. Moreover, the context is emblematic of a modern conflict that is hybrid in nature. The conflict has entailed traditional military combat; Russian misinformation campaigns and cyberattacks; and the threat of chemical, biological, radiological, and nuclear (CBRN) attacks [2]. Since the nature of the conflict is multidimensional, disaster preparedness and response in this context must be as well [3, 4].

These dynamics constitute the contextual backdrop for an event, funded by the NATO Science for Peace and Security Programme (G5663), that Dr. Timothy B. Erickson, (Brigham Health, Harvard Medical School, and Harvard Humanitarian Initiative, Boston, MA, USA) and Dr. Sergii A. Ryzhenko (chief physician at Regional Clinical Hospital, I.I. Mechnikov in Dnipro, Ukraine) co-chaired in Dnipro, Ukraine on November 11-15, 2019. The event, “Emerging Technologies and Countermeasures to CBRN Agents: Advanced Training Response to Conflict and Security Challenges in East Ukraine,” was held at Mechnikov Hospital, Oles Honchar Dnipro National University, and the Menorah Hotel. Over 1,000 participants convened for panel discussions, didactic lectures, and an advanced training on various dimensions of disaster response.

The agenda for the event built upon earlier expert discussions conducted at an advanced research workshop also funded by the NATO Science for Peace and Security Programme (G5432) and co-chaired by Dr. Erickson and Dr. Mykola Prodanchuk from the Ukrainian Ministry of Health’s Research Centre of Preventive Toxicology, Food and Chemical Safety. This earlier event—held in Kyiv, Ukraine in May 2018—brought together key academic leaders and policymakers in NATO member and partner countries to synthesize expert knowledge on topics of environmental health and security in Ukrainian conflict zones and to develop a policy-oriented research agenda on this issue [5].

Informed by the 2018 discussions, three main prongs were planned for the 2019 event in Dnipro. The first prong focused on capacity building and facilitating interoperability between international, national, and local actors engaged in disaster preparedness and response in Ukraine. The second prong focused on opportunities and risks of emerging technologies in disaster response. The third prong focused on the role of women in Science, Technology, Engineering, and Mathematics (STEM) [6]. This report provides an overview of the key discussions and outcomes of the event.

## International, National, and Local Capacity Building and Interoperability

Participants discussed various issues related to international, national, and local capacity building and interoperability, with a focus on CBRN issues but also addressing disaster preparedness and response more broadly in Ukraine. One issue is human capacity, and participants highlighted the need for a greater, sustained investment in training.

This event included a capacity building dimension. In addition to the thematic discussions that occurred, at this event, over five hundred participants and international medical and military actors attended training sessions on five emergency response themes: 1) CBRN response; 2) basic emergency care (a course developed by the World Health Organization on how to manage acute life-threatening conditions with limited resources); 3) point-of-care ultrasound; 4) remote damage-control resuscitation; and 5) all hazards disaster response (which encompassed natural disasters and infrastructure failings, fires and radiological events, pandemics, active shooter incidents, and other mass casualty events). These hands-on sessions followed a “Train the Trainer” model, aiming to produce a network of expert trainers in Ukraine who can themselves use these training methodologies to further capacitate emergency responders across the country [7–9].

Additionally, Ukrainian students and academics attended strategic thinking sessions centered around SWOT analysis (strengths, weaknesses, opportunities, threats). At these sessions, attendees discussed their concerns for their lives, careers, and the future of the country as a whole, and how they could think about these issues in a more strategic manner. The SWOT analysis sessions offered an opportunity for attendees to explore ways to empower themselves to think, process and plan strategically to achieve their objectives.

A second issue that participants discussed is the structures and resources in place to facilitate interoperability at various levels (local, national, and international) of emergency response. Participants noted that evacuation, for example, is not simply a matter for emergency responders and volunteers, but for the state in terms of coordination, providing resources, and ensuring that adequate infrastructure is in place. Participants highlighted Mechnikov Hospital as a success story. In the wake of the armed conflict that erupted in 2014, the hospital scaled up and transformed itself into a trauma response center that treated soldiers and civilians wounded in the fighting. Dnipro Military Hospital also successfully scaled up during this time period. However, at the oblast and national levels, there is a sometimes chaotic competition for resources and little coordination. Participants also discussed—even in spite of international investment in emergency response in Ukraine—a need for greater resources from the international community to feed into efforts to boost capacity building and promote interoperability.

## Emerging Technologies

Noosphere, an non-government organization focused on scientific research and information provision, co-sponsored sessions during which experts, entrepreneurs, and students from Ukraine and across the globe discussed the role of emerging technologies—including virtual reality, unmanned aerial vehicles (UAVs), and digital real-time mapping—related to emergency response. Speakers hailed from a wide array of technology companies, including Philips Healthcare, FUJIFILM SonoSite, Graftworx, 3D Systems, My Pol System, Trident Defence, TipTags, Nightingale Security, SoftServe, and Limpid Group.

Participants discussed recent technological innovations and the practical applications of new tools. Examples that participants discussed include hazard prevention (artificial intelligence that can recognize when people entering a hazardous zone are not wearing appropriate personal protective equipment), medical response (an obstetrical medical device for autotransfusion in post-partum haemorrhage), and humanitarian aid delivery (the use of UAVs) [10]. It is clear that emerging technologies are crucial for emergency preparedness and response. However, participants also examined the challenges of developing new technologies, including the ability to secure funding for innovations with an uncertain success. Additionally, there can be risks in implementation that are important to mitigate. For example, the use of unmanned aerial vehicles (UAVs) in emergency response has become widespread but remains controversial, because they remove people and accountability from response structures.

## Women in Science, Technology, Engineering, and Mathematics

A half-day session held at Oles Honchar Dnipro National University focused on the role women in STEM, giving participants the opportunity to address issues of gender diversity in STEM, as well as gender discrimination dimensions of emerging technologies. Female experts in STEM from universities across the globe—including Dnipro National University, Harvard University, Leiden University, and the University of California, San Francisco—engaged in this session.

Participants shared their observations of the ways in which gender discrimination persists in STEM disciplines, as well as how women and men can work together toward gender equality with emerging technologies [6]. Speakers shared experiences of their own personal experiences facing gender discrimination. An important point raised was that different generations, and different cultures, experience gender discrimination in various ways: ranging from overt bias to more subtle, implicit discriminatory practices. Participants also addressed gender discrimination in new technologies, such as implicit biases that make their way into algorithms, with implications for a wide range of issues, including the job market and credit scores. In light of the importance of STEM for disaster response, as well as the need for maximizing the potential for human capital, addressing gender diversity in STEM will need to be a part of ongoing and future emergency response capacity building efforts.

## Concluding Remarks

The overall takeaway from this multi-faceted event entails three particular dimensions of emergency preparedness and response in Ukraine. The first dimension is resources. A sustained and expanded investment in this area from local, national, and international actors is necessary. Although there are success stories—for example, the scaling up of Mechnikov Hospital and Dnipro Military Hospital in 2014—this area remains resource scarce. International participants, including a speaker from the Organization for Security and Co-operation (OSCE) in Europe, mentioned that their own persistent resource limitations have hindered their ability to engage as deeply in Ukraine as they would wish. This dimension also encompasses human resources, including the need to continue to build the pool of trained emergency responders in Ukraine. The second dimension is structures for interoperability. Coordination of preparedness and response efforts is necessary to maximize the impact of investments made emergency preparedness and response. A third dimension is leadership. Coordination at the institutional level requires leadership qualities at the individual level in terms of management skills, strategic thinking, and innovative creativity. Capacity building efforts should also focus on this element. A forward-looking vision will focus not only on immediate emergency response needs but also on an inter-generational effort to cultivate a pool of future emergency response leaders in East Ukraine capacitated to meet the need of tomorrow.

## Data Availability

**Link to meeting agendas:** https://natospsdnipro.noosphere.events/ https://www.facebook.com/HarvardUkraine
